# Deep in situ microscopy for real-time analysis of mammalian cell populations in bioreactors

**DOI:** 10.1038/s41598-023-48733-x

**Published:** 2023-12-12

**Authors:** Jean-Sébastien Guez, Pierre-Yves Lacroix, Thierry Château, Christophe Vial

**Affiliations:** 1 Université Clermont Auvergne, Clermont Auvergne INP, CNRS, Institut Pascal, 63 000 Clermont-Ferrand, France; 2 Logiroad.AI, 63 178 Aubière, France

**Keywords:** Machine learning, Optical imaging, Cell death

## Abstract

An in situ microscope based on pulsed transmitted light illumination via optical fiber was combined to artificial-intelligence to enable for the first time an online cell classification according to well-known cellular morphological features. A 848 192-image database generated during a lab-scale production process of antibodies was processed using a convolutional neural network approach chosen for its accurate real-time object detection capabilities. In order to induce different cell death routes, hybridomas were grown in normal or suboptimal conditions in a stirred tank reactor, in the presence of substrate limitation, medium addition, pH regulation problem or oxygen depletion. Using such an optical system made it possible to monitor real-time the evolution of different classes of animal cells, among which viable, necrotic and apoptotic cells. A class of viable cells displaying bulges in feast or famine conditions was also revealed. Considered as a breakthrough in the catalogue of process analytical tools, in situ microscopy powered by artificial-intelligence is also of great interest for research.

## Introduction

Biopharmaceuticals refers to drugs produced in biotechnological processes using living cells, as antibodies, peptides and recombinant proteins. Their high specificity and potency, compared to other compounds, arise mainly from their macromolecular composition, provided by a structural complexity that is required for specificity^1^, which also makes biopharmaceuticals challenging to produce and characterize^2^. Sizing of the biopharmaceutical market shows some discrepancies in the reported values but almost all projections converge to a strong increase in the annual growth rate, around 10%, until 2030. Among the important driving factors of this market growth, certain could be accelerated in regard to provisional management of pandemics. In the world, the total culture and fermentation capacity for biopharmaceuticals production represented 15.0 million liters in 2017, with 70% of these facilities displaying large-scale capacities of more than 500 L, and 62% referring to animal cell cultures^3^. Nevertheless, the tremendous demand for these complex drugs involves continuous improvements to robustify, extent and intensify their production.

In these cultures, process parameters represent mean values that do not express the behavior and the heterogeneity at the individual cell level. Cells are indeed subjected to different types of environmental stresses like hydrodynamic shearing or concentration gradients, exposing them to varying external conditions of dissolved oxygen, dissolved carbon dioxide, pH, nutrients and by-products, that affect their physiology^4^, and is amplified when the operation is performed in large-scale bioreactors. To study the cell-to-cell variability caused by this heterogeneity, which is defined as an extrinsic heterogeneity, various types of scale-down simulators are developed, tending to simulate at lab-scale the heterogeneous environments of industrial bioreactors^5,6^. But they are not yet as widely used for mammalian processes as they are for microbial processes, since the setup of such systems is more challenging for mammalian cells. Transferring the knowledge on the extrinsic heterogeneity gained at a small scale to production scale should also be completed with the insights obtained on the intrinsic heterogeneity caused by the stochasticity of both gene expression and metabolic reactions, at single-cell level^7,8^. As heterogeneity globally affects isogenic cell lines, it raises the importance of developing new methods for bioprocess development and monitoring, that correlates cell cultivation and cell-to-cell analysis^9^, preferably real-time.

The environmental stresses experienced by the cells within a bioreactor lead to an increase in the number of dying or dead cells, which is directly linked to product titer and quality^10^. Until recently, three routes of cell death were defined according to distinctive morphological criteria, namely apoptosis, autophagy (that can serve either pro-survival or pro-death functions) and necrosis^11,12^. Apoptotic cells expose phosphatidylserine on the cellular surface at the initial or early apoptosis stages, followed by plasma membrane blebbing, chromatin condensation, nuclear fragmentation, cytoplasmic shrinkage, and culminate with the formation of small vesicles known as apoptotic bodies. Autophagic cells mainly manifest extensive cytoplasmic vacuolization. Necrotic cells principally display cytoplasmic swelling and rupture of plasma membrane, in late stages. Since then, the research in the field expanded continuously, revealing numerous other regulated cell death routes^13^, showing cross-talking and balanced interplay^14,15^. Nowadays, morphological classification of cells within in vivo applications is no longer intended to exist without molecular analysis (genetic, biochemical or immunologic), which are obligatory for a deepened comprehension of cell function and physiology. In the case of bioprocess applications, which are mainly represented by in vitro cultures of less complex unicellular forms, morphological classification can be considered more easily without further molecular analysis, as long as a correlation can beforehand be established with the physiology and the function of the cell.

Accidental cell death resulting in the disassembly of the plasma membrane can occur when a major bioprocess failure is encountered (extreme physical or chemical conditions), usually followed by a process shutdown. But in the vast majority of cases, environmental factors impacting cell death gradually build up. In cell culture systems, it was early reported that apoptosis was the major route of cell death^16,17^, confirmed since then^18^. Although apoptosis plays a major role, necrosis may also be observed^19–21^. Considering autophagy, just a few studies were reported^22,23^.

Closely linked to cell death, viability is a critical key parameter of the process that can be monitored either with applying online/inline (sensor inside the bioreactor, no sampling) or atline (sensor outside the bioreactor, with automated sampling) techniques. According to the regulatory boundaries set by the European Medicines Agency or the U.S. Food and Drug Administration, monitoring is necessary to document repeatability as quality control and ensure a proper operation regime. For this purpose and further research applications, numerous process analytical tools exist^24^. Online monitoring of viability is considered with using dielectric^25^, UV/VIS, fluorescence^26^, IR, and even Raman or NMR spectroscopies^27^. But no studies achieving heterogeneity and cell population studies based on individual cell analysis for bioprocess monitoring has been reported with these techniques. In situ microscopy (ISM) could have the potential to analyze more precisely cell populations through cell imaging but the reported studies only estimated viability based on grey value dispersion criteria^28^ and entropy state^29^ of individual cells. Based on ISM, flow morphometry was reported recently to establish the quality of red cells^30^, but no population analysis was carried out to estimate cell viability, let alone for a use as an online process monitoring tool of death cells. Atline flow cytometry (fully-automated for sample preparation) allows to establish population analysis^20^, but is complex to operate. It can be combined with other techniques, as electrical impedance^31^ or fluorescence microscopy^32^. Imaging flow cytometers allow to detect and prepare images of individual cells analyzed from the bulk cell population. Lab-on-a-chip microfluidic-based systems also represent important methods to examine in detail cellular differences, with the advantage to achieve partial or full spatio-temporal resolution, when compared to cytometry^9^. To date, these techniques run atline or offline, and such cytometry-based systems operate only in specialized laboratories, at speeds that are not compatible with short-lived dynamic phenotypes^33^, as encountered in bioprocess monitoring. Time-lapsed imaging should also be mentioned as it allows interesting online single-cell resolution^34^, but this technique is not applicable to the monitoring of planktonic cultures of cells grown in bioreactor, only static ones in perfusion flow bioreactor^35^.

Taken together, it appears that no simple technique yet exists for accurate individual cell analysis and non-invasive online monitoring of cell population in bioreactor.

However, advances in optical microscopy and cell cultivation have led to the setup of image analysis methods allowing to extract quantitative information from vast data sets. The relevance of using machine learning and computer vision approaches have been highlighted by numerous works^36–39^. As cell death has significant impact in cell cultures in bioreactor, upstream process development should account for cell death monitoring, and propose strategies to avoid it as much as possible, rather than trying to revert it^18,40^.

Taking up the challenge to propose a new tool for the study of heterogeneity during cultivation processes in bioreactor, at the industrial scale or for research purposes, this work investigates a new non-invasive online monitoring technique based on an in situ imaging microscopy coupled to convolutional neural networks (CNN).

## Results and discussion

### Labelling of cells extracted from ISM micro-photographs

The starting point of this work consisted in the thorough examination of a 4000 images database.

Collected images (bitmap, 8 bit) have a size of 1392 × 1040 pixels, corresponding to 6.6 × 8.8 mm^2^ (available in the CNRS Research Data repository). A part of the cellular objects contained in the ISM-image database were far from the focus plane and appeared as blurred objects, and therefore not considered in the sequel of this work. For sharp cells that belonged to the focal plane, slight variations of the exposure of the ISM-cell images could be observed. Cells located between the illumination diode and the focus plane could act as lenses leading to brighter spots while the other ones, which were located behind the focal plane, had a darker appearance. Hybridoma cells contained in ISM-images were BalB/c murine lymphoid cells fused with murine myeloma cells. In these working conditions, six classes of cells could be presented (Table 1) and compared to previous works^41,42^. Visual inspection and analysis of the surface representation of the ISM-cell images belonging to class 1 exhibited mainly a pseudo-circular shape and a homogeneous texture. These features are commonly associated to healthy and viable cells. The evidence of this correlation was given in previous works based on imaging flow cytometry^19,43^. It was also shown with comparing cell images from ISM and epifluorescence microscopy after differential staining with calcein AM and ethidium homodimer^44^. It is worth noting that viable cells could also occur as twins in the case of mitotic event (class 6).

**Table 1:**
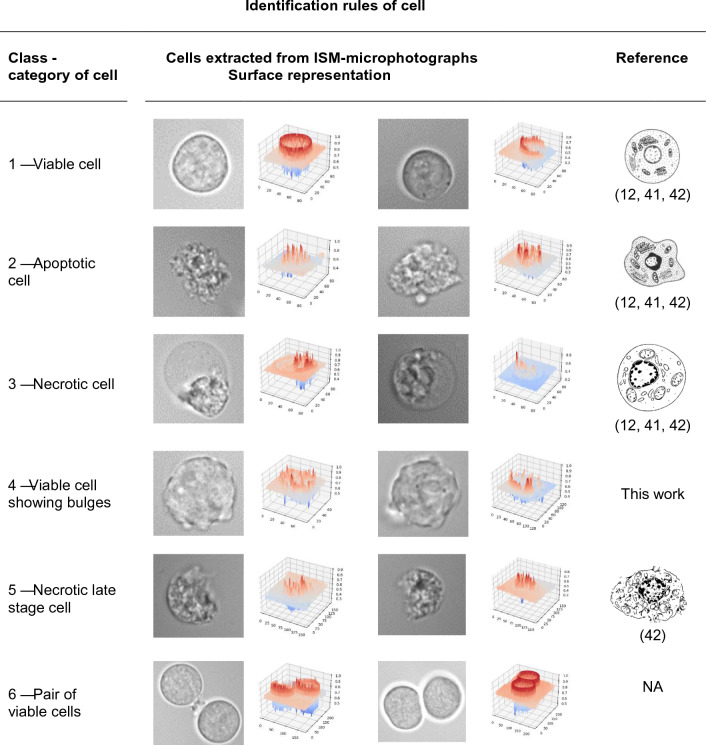
Raw ISM-images of cells extracted from micro-photographs recorded in cell suspension cultures.

Examples of images of individual cells and their surface representations classified in six categories: viable, apoptotic, necrotic, viable displaying bulges, necrotic late stage and pairs of cells.

It is known that live cell imaging shows a loss of smoothness of the cell texture of dying or dead cells, in contrast to viable cells ^19,36,40^. The cell body exhibits more irregular features and enters a stage of inhomogeneity. The roundness of the cell border is also affected. Not viable cells, which are increasingly found in ISM-images corresponding to lower viability values, were partitioned into two main classes. Visual inspection and analysis of the surface representation of cells belonging to class 2 showed strong variations in the texture of the cell body and a lack of roundness of the cell contour. These cells appeared inhomogeneous with irregular and shrunken cellular features. Their morphological pattern revealed high similarity to cells previously classified as apoptotic cells^12,19,43^. A part of the cells belonging to class 2 also showed small vesicles, which could correspond to apoptotic bodies. As for cells belonging to class 2, cells belonging to class 3 displayed inhomogeneity. They exhibited changes in the intracellular morphology when compared to viable cells. Their morphological pattern was associated to the almost perfect roundness of their appearance, which occurs during cytoplasmic swelling. This feature is well-known and allows to distinguish necrotic cells from others^12,19,21,43^. Close to this morphological pattern based on roundness and inhomogeneous texture of the cell body, few cells showed a distinctive blurred area associated to the discontinuity of the cell border. These morphological features could be associated to the rupture of the plasma membrane that occur after the cytoplasmic swelling stage^11^. These cells were attached to class 5 and were labelled as late-stage necrotic cells. Hybridoma cells studied in this work could thus be classified in at least 5 different classes. Similar cell morphological features were already shown on ISM-images for other proprietary hybridoma and Jurkat (DSMZ ACC 282) cell lines^29^. As this classification was obtained according to well-known morphological features of mammalian cells^41^, it should be possible to apply to different types of cells.

In the 4000 images database, it was also possible to observe a particular cell pattern with a slightly less homogeneous texture, as well as a less circular contour, compared to viable cells belonging to classes 1 and 6. This visual image inspection was confirmed by the surface representation of the cells. At this stage of the work, it was hard to establish a clear relationship between this distinctive cell pattern and previous bibliography. As these cells showed typical bulges at their surface, they were simply labelled as “viable cells displaying bulges” in the learning database (class 4).

To summarize, the ISM-cell images belonging to class 1 and 6 corresponded to viable cells (single and pair), class 2 to apoptotic cells, class 3 to necrotic cells, class 4 to viable cells displaying bulges and class 5 to necrotic late-stage cells.

### Implementation of the AI-ISM cell classifier and application to a bioprocess

**Figure 1:**
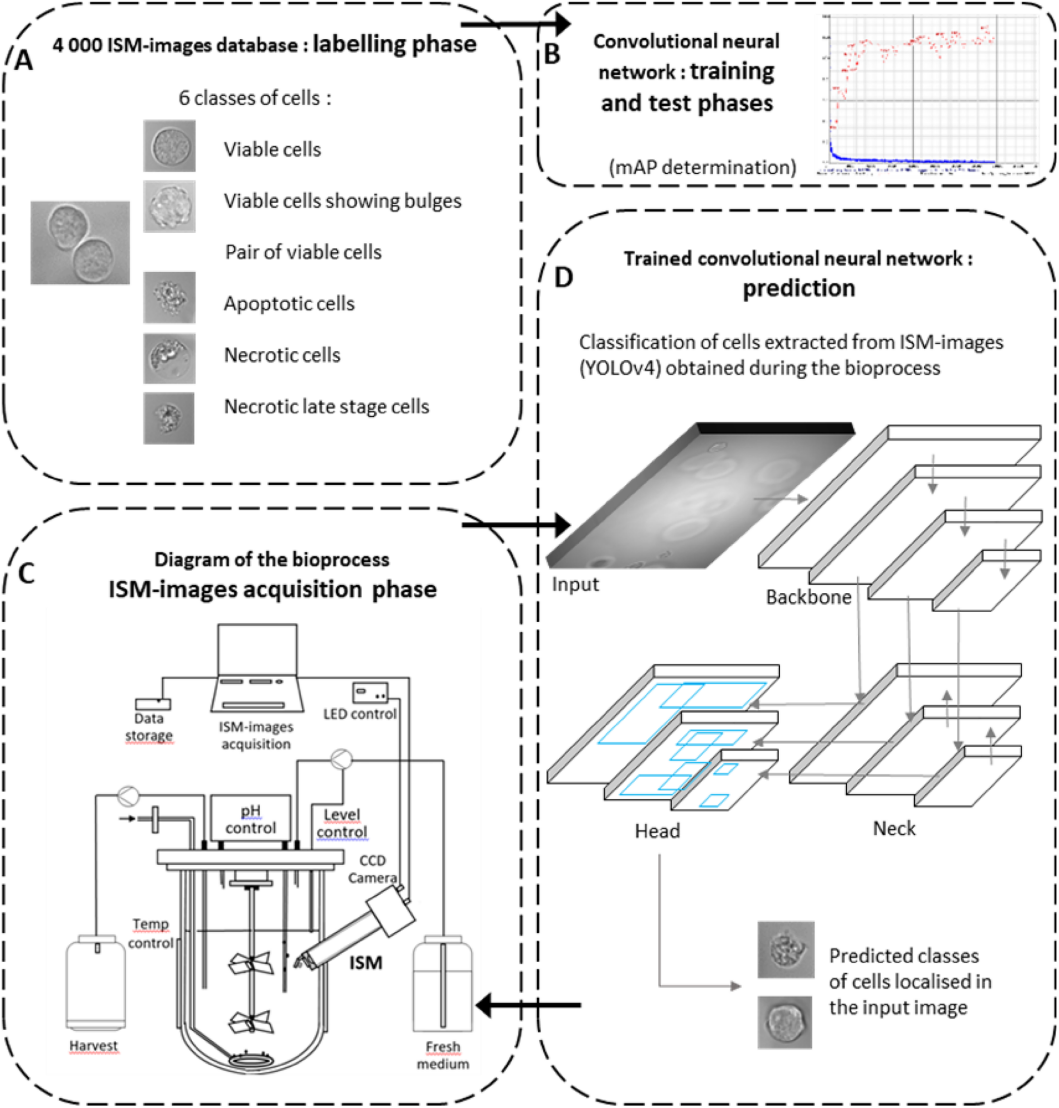
Overview of the bioprocess control and monitoring and implementation of the CNN-based classifier. (**A**) Set up of a labelled database obtained thanks to the annotation of 4000 representative images taken by ISM. (**B**) Training and test phases of the model. Evaluation of the performance of the object detection algorithm is done with optimizing its mean average precision (mAP). (**C**) Diagram of the ISM mounted on a bioreactor for the observation of cells suspended in a nutrient medium during a biopharmaceutical production process. LED-flash illumination generates still images of cells drifting by the quartz window in front of the ISM objective. The images showing cells with different morphological patterns are acquired on-line during the bioprocess. (**D**) The prediction of the different classes of cells localized in the images is done on-line by the trained and tested convolutional neural network. The evolution of the concentration of each class of cells is used for process monitoring and control.

As shown in Fig. 1A, the labelling of a database made up of 4000 images was done according to the identification rules of cellular objects presented in Table 1. Objects localized at the periphery of the ISM-image were not labelled when less than half of the cell body was visible. In the case of overlapping cells, a seldom event, it was chosen not to label the cells. In these conditions, 907 cells belonging to the six different classes of cells observed in the 4000 images were labelled: (class 1—viable cell): 231; (class 2—apoptotic cell): 260; (class 3—necrotic cell): 106; (class 4—viable cell displaying bulges): 262; (class 5—necrotic late stage cell): 19; (class 6—pair of viable cells): 29. A set of 3400 images taken from the annotated database was used for the training phase of the object detection algorithm. A set of 600 images was then devoted to the test phase of the model allowing to measure its performance for each class of cells, measured through the determination of a mean average precision (mAP) (see Fig. 1B). Two different strategies of training of the model were used: with or without pooling together (class 1—viable cells) and (class 4—viable cells with bulges). When pooling classes 1 and 4, the mAP value of the corresponding class was equal to 95.7%, expressing a remarkably high percent of correct detection of individual viable cells, with or without bulges, by the model. The second training of the model was done independently from the first one. In these conditions, the mAP value of class 1 and class 4 cells could be calculated separately, 67.0% and 66.9%, respectively. It could be specified that the mAP value for the other cell classes (apoptotic, necrotic and necrotic late stage and pair of cells) was 73.0%, 79.9%, 80.9% and 85%, respectively. The test/train ratio for these classes was 16, 15, 17, 15, 21 and 14%, respectively.

A deepened analysis was done on a reduced set of 140 images, to compare separately labelled and predicted results for (class 1—viable cells) and (class 4—viable cells with bulges). The comparison showed an error of 25% for (class 1—viable cells) and 27% for (class 4—viable cells with bulges). The total number of (class 1—viable cells) and (class 4—viable cells with bulges) differed only slightly when comparing labelled and predicted classes, respectively 2.5% and 5.4%. It thus appeared that the error made on (class 4—viable cells with bulges) predictions was mainly due to a confusion with labelled (class 1—viable cells), and vice-versa. It could be easily explained by the morphological resemblance between these two classes, particularly within the transient states between those two classes, which made it more difficult to distinguish one from the other. It was therefore important for further online population analysis to keep in mind that the precision of the prediction of (class 1—viable cells) and (class 4—viable cells with bulges) could be affected because of differences in morphological features that are less marked between these classes. Cells were cultured as presented on the bioprocess diagram described on Fig. 1C. On line object detection consisted in localizing objects in ISM-images. Neural networks used for accurate object detection usually do not operate on line. To address this problem, a YOLO accurate object detector which follows a one-stage method of detection to prioritize inference speed, was used for on line detection on a conventional GPU (37). The automatic classification of the objects localized in ISM-images by YOLO was summarized in Fig. 1D.

### Online monitoring of cell population during bioprocess

#### Overview of the bioprocess

**Figure 2:**
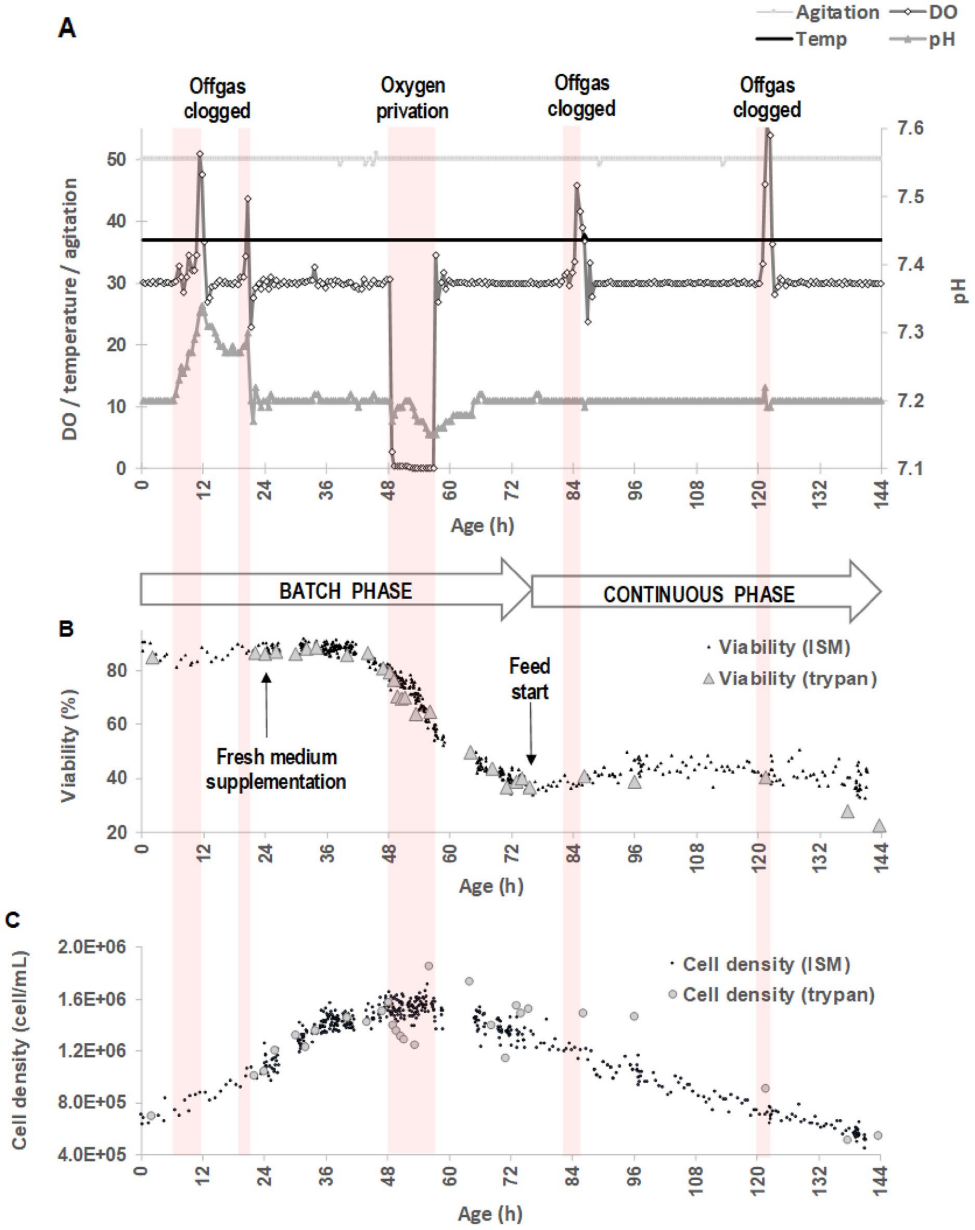
Example of a culture of BalB/c murine/murine hybridoma cells for the production of monoclonal antibodies in bioreactor. (**A**) Online monitoring of the agitation speed (RPM), dissolved oxygen (DO), temperature (°C) and pH. Process is operated in a batch mode for 75,5 h and in a continuous mode between 75,5 and 145 h. Main process events are given (offgas clogging/pH control failure, medium supplementation, stopping of the oxygen and feed start). Evolution of the viability expressed in % (**B**) and cell density expressed in cell/mL (**C**) measured online by AI-ISM and offline by trypan blue dye exclusion assay.

A representative culture of cells producing biopharmaceuticals was studied. The evolution of online parameters as a function of the agitation speed (RPM), dissolved oxygen (DO), temperature (°C) and pH are shown in Fig. 2A. The process was first operated in a batch mode for 75.5 h. The cells were then cultured in a continuous mode, between 75.5 and 145 h, at a dilution rate of 0.0125/h. To monitor different patterns of cell population that can occur during a culture in bioreactor, the effect of different process events was analyzed. These events may either belong to the class of unwanted events, leading to suboptimal process conditions, or be the result of an action programmed by the operator, as part of the intended process recipe. An unwanted event that can be encountered often is the blocking of the gas outlet due to the presence of humidity in the outlet filter, which could be observed at 6, 18, 81 and 120 h of growth. Blocking of the gas outlet induced an overpressure in the reactor vessel which first increased the partial pressure of oxygen and thus the dissolved oxygen. If the gas outlet issue was not addressed rapidly, dissolved oxygen then decreased proportionally to oxygen uptake rate of the cells, until the oxygen was totally exhausted. Blocking of the gas outlet could also cause disturbances in pH regulation meant to be controlled by addition of carbon dioxide, as observed between 6 and 21 h of growth It could also be responsible for the reduction or cessation of oxygen inflow. In this experiment, oxygen was completely stopped between 48.25 and 56 h of growth. The cell population patterns were also studied during routine process control actions, as the addition of fresh media (at 24 h of growth) or the start of a continuous media feed (at 75.5 h of growth).

During the bioprocess, a set of 848,192 images was recorded, corresponding to 283,760 detections made by the cell classifier (Fig. 2B and C). Depending on the age of the culture and the related cell density, image taken by the ISM contained between 0.13 and 0.54 detected cells. Each experimental point presented on the curves corresponded at least to a set of 1000 images. Note that during the duration of the bioprocess, the acquisition of ISM-images was interrupted for technical reasons during short periods: [26.8–30.7 h]; [41.6–43.9 h]; [58.8–65.1 h]; [67.1–69.5 h]; [141.1–144.2 h]. It was also observed that the viability measured just after inoculation at 85% was lower than the expected value, around 95%, explained by an excessive time lag between the end of the preculture and the inoculation of the bioreactor, caused by the transfer of the preculture from one location to the other.

#### Cell density and viability correlation between trypan blue dye exclusion test and AI-ISM

The conversion coefficient that allowed estimating the cell density from the number of cells per image provided by the ISM was first determined. The objects detected by the ISM belonged to a virtual probe volume: the optical device and the CCD sensor size defined the lateral borders of the probe volume, while its depth depended on the parameters of the classification process performed by the CNN. The AI-image processing algorithm was indeed recognizing ‘‘cell-classes’’ of only those cells which appeared with sharp contours. The optical depth of field was thus implicitly defined by the algorithm sensitivity with respect to focus ^44,45^. This could be expressed as X(t) = α y(t) where X(t) was the cell density (cell/mL) and y(t) the measurement made by the object detector (cell/image). The conversion coefficient “α” allowed estimating the cell density from the number of cells per image provided by the ISM. It was considered as the ISM calibration parameter (image/mL) and interpreted as the inverse of the ISM reference volume. In the present study, the coefficient “α” was equal to 3.2 10^6^ image/mL. This value belonged to the same scale of magnitude but was higher than the conversion coefficient, i.e. 9.26 10^5^ image/mL, obtained in a previous comparable study^45^. It was caused by surface sizes of the optical device/CCD sensor and image processing strategy (that determines the depth) that were different between the previous and the present work. The regression model applied to the relationship between the cell density measured by trypan blue dye exclusion test and the number of cells per image detected by the ISM gave a value of 0.74, which was lower than the correlation coefficient of 0.99 previously obtained by authors^45^. The difference could be explained as previous results were reported for a bioprocess controlled to reach high cellular concentration keeping high viability values. In the present work, different process events and control failures were induced voluntary to allow the observation of various death cell population patterns while viability strongly decreased. In these suboptimal culture conditions, the precision of the cell counting procedure of the trypan blue dye exclusion test, and thus the relationship with AI-ISM measurements, can be affected^29^.

The cell density of total viable cells measured by AI-ISM was counted as the sum of the cell density of viable cells showing bulges or not, and pair of cells. In parallel, the cell density of total dead/dying cells was counted as the sum of the cell density of apoptotic, necrotic and late necrotic cells. The total cell density (or cell density) was calculated as the sum of the cell densities of total viable and dead/dying cells. As it is usually done, the viability was calculated as the ratio of the cell density of total viable cells upon the total cell density. Thanks to these counting and calculating rules, the viability and the total cell density measured by the AI-ISM were calculated and compared to the results obtained with the trypan blue dye exclusion test (Fig. 2B and C). The mean error between the results obtained with both methods was estimated with calculating a Mean Absolute Percentage Error (MAPE). When considering the entire bioprocess, the MAPE value was equal to 10.88% for total cell density, and 6.95% for cell viability. It is known that the trypan blue dye exclusion test is more precise when used for viability assessments of a cell culture, below 5% error, than for the estimation of cell population density, which can reach an error of 20%^46^. This explained why the MAPE value calculated for the viability parameter was lower than that calculated for the cell density parameter. Interestingly, estimates obtained for early stages of the bioprocess when viability was higher than 80%, from inoculation until 48.25 h of growth, reduced MAPE from 10.88 to 6.70% for cell density and from 6.95 to 1.68% for viability. This is one of the results to be highlighted in the present work. It also could be pointed out that viability estimates obtained for viability values higher than 28% still reduced MAPE from 6.95 to 3.87%, which should also be highlighted. Conversely, viability values lower than 28% drastically increased the MAPE, from 6.95 to 50.17%, indicating that in this range of viability values, it was no more possible to correlate the results of both methods. Although trypan blue exclusion assay is the traditional method for measuring cell viability, it has several potential drawbacks, particularly at low viability^47^. As cells begin to die, dying cells can indeed show diffuse light blue patterns when stained with trypan blue, which may be missed during manual cell counting. To assess this phenomenon, the variation coefficient of the trypan blue dye exclusion assay was determined experimentally for low viability. A variation coefficient of 18.80% was measured for a mean viability of 27% (quadruplicate analysis). Additionally, the number of cells counted on the haemocytometer for very low viability samples can be low, so that a few mismatches of dying/dead cells strongly influence the evaluation of cell viability. For similar reasons, viability estimates made by AI-ISM showed a more scattered distribution, particularly at the end of the bioprocess after 120 h of growth.

Considering cell density estimates, the MAPE increased from 10.88% for the overall bioprocess to 14.98% when only considering the [48.25–53 h] interval. During this period of time, the bioprocess encountered both a substrate limitation, which began at 40 h of growth, and a severe lack of oxygen. Estimates made by AI-ISM showed less noisy and surprisingly, consistently higher values than measurements made by the trypan blue dye exclusion test. This could be explained by the difficulties encountered by the operator in analyzing samples taken during a period that corresponded to a strong acceleration of the kinetics of cell death, when some of the cells observed on the haemocytometer grid became smaller^29^ and more shriveled than usual, which ultimately leads to counting errors. Later on in the bioprocess, and until the end, when values of viability inferior to 60% were reached, higher counting values were obtained by the trypan blue dye exclusion test compared to AI-ISM method. The counting appeared to be even more difficult during this period as some trypan blue—stained cells exhibited a blurred aspect and more non-uniform morphological characteristics, increasing the uncertainty of the counting. This is indicative of the poor reliability of the trypan blue dye exclusion test when it is used to estimate the total number of cells in a culture, especially for samples that correspond to low viabilities values.

However, taken together these results indicated the usefulness of AI-ISM cell density and viability estimates for routine bioprocess monitoring, allowing high accuracy in the evaluation. It also revealed a strong robustness when applied to a culture where viability fluctuations cover a wide range.

#### Deepened analysis of cell population by AI-ISM

**Figure 3:**
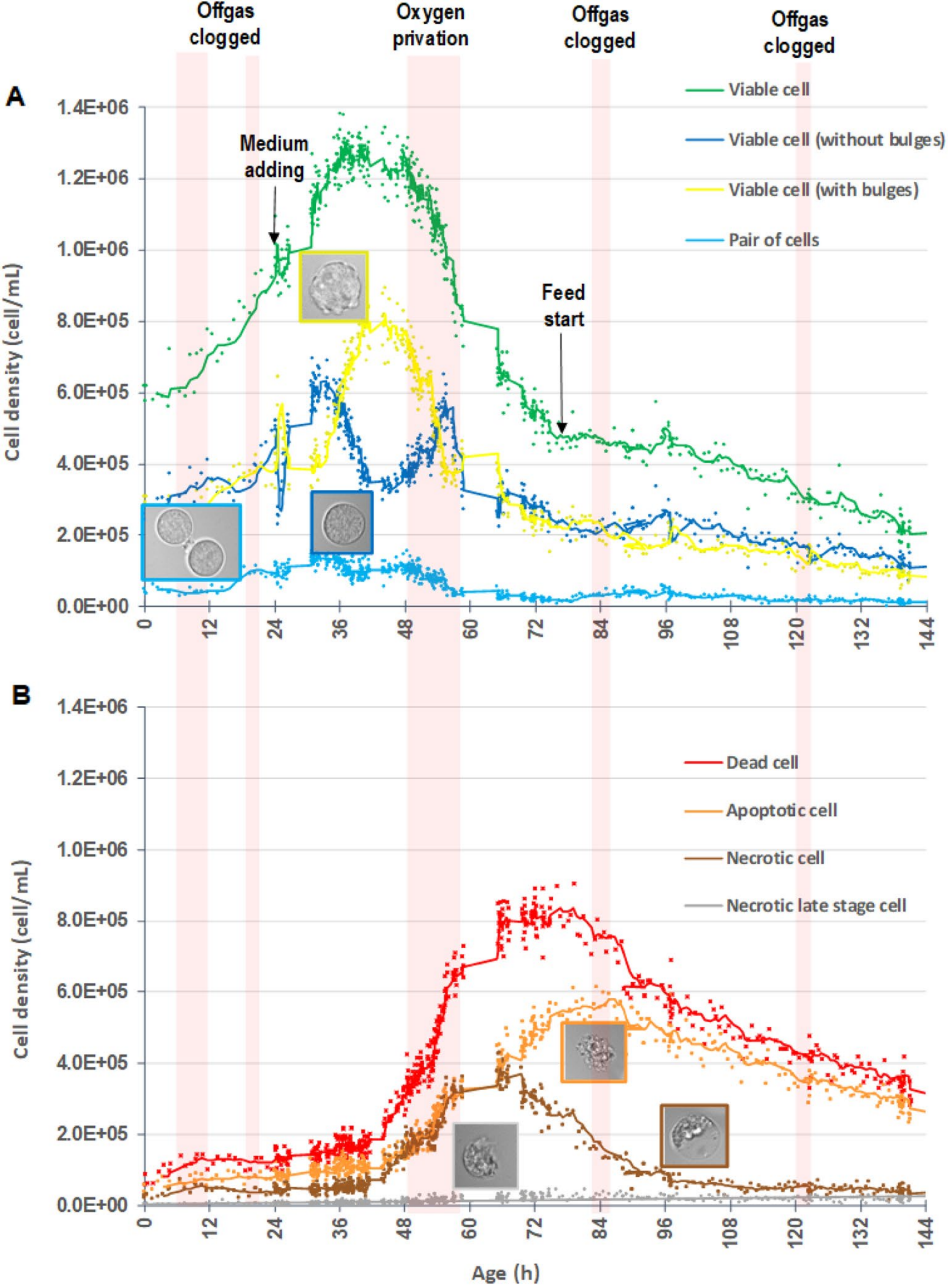
(**A**) Evolution upon culture time of the cell density (cell/mL) of total viable cells (), viable cells without bulges (), viable cells with bulges () and pair of cells () measured by AI-ISM. Raw ISM-images extracted from micro-photographs show the different morphological pattern of the three types of viable cells (viable, viable with bulges and pair) are given. (**B**) Evolution upon culture time of the cell density (cell/mL) of total death (), apoptotic (), necrotic () and necrotic late stage () cells measured by AI-ISM. Raw ISM-images extracted from micro-photographs displaying the different morphological pattern of the three types of death cells (apoptotic, necrotic and necrotic late stage) are given. Main process events are given (offgas clogging/pH control failure, medium supplementation, stopping of the oxygen and feed start).

As detailed in the previous section, the cell density of total viable cells measured by AI-ISM was counted as the sum of the cell density of viable cells showing bulges or not, and pair of cells. Figure 3A. shows the evolution of the cell density of the three classes of viable cells. The cell density of total dead/dying cells was counted as the sum of the cell density of apoptotic, necrotic and late necrotic cells. Figure 3B shows the evolution of the cell density of each class of dying/dead cells. It was highlighted in the previous paragraph that using these counting rules allowed to estimate (or measure) cell density and viability with high accuracy. By extension, it was assumed that the counting of cells belonging to these six classes was correctly done by the AI-ISM. For a thorough analysis of the kinetics of each cell classes upon time, Table 2 presents the relative change in the number of cells belonging to each cell classes for nine identified periods that corresponded to bioprocess events, observed at different stages of the cell growth: gas blocking and related pH control failure, medium supplementation, stopping of the oxygen and feed start. To better decipher the trends during the bioprocess, the curves of different classes of cells that were detected were smoothed with applying a moving average.

**Table 2:** Cell density (expressed in cell/mL) measured by AI-ISM at the beginning and at the end of nine different process events.

Process event/Growth phase		Cell density measured by deep ISM (cell/mL) and relative change (%)					
	Age (h)	Viable	Viable with bulges	Pair	Apoptotic	Necrotic	Necrotic late stage
Gas blocking 1&2	2	2.67 10^5^	2.56 10^5^	2.88 10^4^	6.64 10^4^	2.53 10^4^	3.16 10^3^
Lag and exponential growth phase	22	3.85 10^5^	3.60 10^5^	9.79 10^4^	8.10 10^4^	4.00 10^4^	6.38 10^3^

		+ 44%	+ 41%	+ 240%	+ 22%	+ 58%	+ 102%
Fresh medium addition (early effect)	24	3.95 10^5^	3.69 10^5^	9.95 10^4^	8.25 10^4^	4.10 10^4^	7.10 10^3^
Exponential growth phase	26	2.76 10^5^	5.64 10^5^	1.01 10^5^	9.00 10^4^	4.35 10^4^	7.20 10^3^

		− 30%	+ 53%	+ 2%	+ 9%	+ 6%	+ 1%
Fresh medium addition (late effect)	26	2.76 10^5^	5.64 10^5^	1.01 10^5^	9.00 10^4^	4.35 10^4^	7.20 10^3^
Exponential growth phase	27	4.81 10^5^	3.84 10^5^	1.11 10^5^	8.22 10^4^	4.11 10^4^	7.10 10^3^

		+ 74%	− 32%	10%	− 9%	− 6%	− 1%
Substrate limitation	34	6.09 10^5^	4.05 10^5^	1.25 10^5^	1.01 10^5^	4.09 10^4^	6.50 10^3^
Decelerating and early stationnary phase	44	3.45 10^5^	7.81 10^5^	1.01 10^5^	1.03 10^5^	7.60 10^4^	1.52 10^4^

		− 43%	+ 93%	− 19%	+ 2%	+ 86%	+ 134%
Substrate limitation	44	3.45 10^5^	7.81 10^5^	1.01 10^5^	1.03 10^5^	7.60 10^4^	1.52 10^4^
Late stationary and decline phase	48.25	7.45 10^5^	3.62 10^5^	1.03 10^5^	1.52 10^5^	1.43 10^5^	1.70 10^4^

		+ 116%	− 54%	+ 2%	+ 48%	+ 88%	+ 12%
Oxygen depletion/substrate limitation	48.25	7.45 10^5^	3.62 10^5^	1.03 10^5^	1.52 10^5^	1.43 10^5^	1.70 10^4^
Death phase	56	5.40 10^5^	3.80 10^5^	4.90 10^4^	3.00 10^5^	3.00 10^5^	2.00 10^4^

		− 28%	+ 5%	− 52%	+ 97%	+ 110%	+ 18%
Oxygen resupply/substrate limitation	56	5.40 10^5^	3.80 10^5^	4.90 10^4^	3.00 10^5^	3.00 10^5^	2.00 10^4^
Death phase	68.25	2.58 10^5^	2.12 10^5^	9.00 10^3^	5.30 10^5^	2.58 10^5^	4.50 10^4^

		− 52%	− 44%	− 82%	+ 77%	− 14%	+ 125%
Starting of the continuous feeding phase	75.5	2.58 10^5^	2.12 10^5^	9.00 10^3^	5.30 10^5^	2.58 10^5^	4.50 10^4^
Transitory phase	86	2.23 10^5^	1.98 10^5^	4.00 10^4^	5.79 10^5^	1.42 10^5^	3.00 10^4^

		− 14%	− 7%	+ 344%	+ 9%	− 45%	− 33%
Continuous feeding phase	86	2.23 10^5^	1.98 10^5^	4.00 10^4^	5.79 10^5^	1.42 10^5^	3.00 10^4^
(no steady state reached)	143.5	9.10 10^4^	8.30 10^4^	9.00 10^3^	3.02 10^5^	3.80 10^4^	1.30 10^4^

		− 59%	− 58%	− 78%	− 48%	− 73%	− 57%

Relative change of cell density (expressed in %) calculated within the duration of a process event.

During the [2–22 h] period, the relative change showed an increase in the total number of viable cells. Particularly, the number of pair of cells increased strongly, i.e. + 240%, which can be explained as cells were mainly grown exponentially during this period, with increased number of mitotic events. Surprisingly, relative changes in the same period also showed an increase in the total number of dead/dying cells, which was not really expected during an early exponential growth phase. Figure 3B shows that this increase was more specifically observed before 12 h of growth. It could be explained by the usual stress that can occur during the inoculation of the bioreactor combined to the blocking of the gas, that occurred between 6 and 12 h of growth, followed by a pH control failure, as the addition of CO_2_ was no more effective. After 12 h of growth, the pH regulation gradually returned to normal as shown in Fig. 2A. The total number of dead/dying cells remained constant between 12 and 22 h of growth. Interestingly in this period of time, a slight increase in the number of apoptotic cells and decrease in necrotic cells was shown (Fig. 3B).

At 24 h of growth, a volume of 200 mL of DMEM supplemented with 4 mM l-glutamine and 4.5 g/L glucose + 10% heat inactivated fetal calf serum was added to the cell culture. The evolution of the kinetic of the total density of viable cells appeared not to be affected during the ten hours that followed this addition (Fig. 2C). By taking a careful look at the AI-ISM data (Fig. 3A), an unexpected effect of this addition could be measured between 24 and 26 h of growth, which clearly showed a strong decrease in the number of viable cells without bulges (− 1.19 10^5^ cells) in favor of the number of viable cells with bulges (+ 1.95 10^5^ cells). In such a surprising way, this phenomenon was followed by a rapid return to normal: between 26 and 27 h of growth, the number of viable cells without bulges increased again (+ 2.05 10^5^ cells), at the expense of the number of viable cells with bulges (− 1.80 10^5^ cells). It could be noted that during the [24; 27 h] period, the number of dying/dead cells remained constant, indicating the absence of deleterious effect of the fresh medium added in the cell culture.

Between 34 h and 48.25 h of growth, the evolution of the number of total viable cells showed a deceleration which could be physiologically explained by the end of the exponential growth phase and the subsequent onset of the stationary phase, caused by substrate limitation. During the early steps of this period [34; 44 h], the number of viable cells without bulges strongly decreased (− 2.64 10^5^ cells) in favor of viable cells displaying bulges (+ 3.76 10^5^ cells). This phenomenon was completely inversed in the late step of this period [44; 48.25 h]: the number of viable cells without bulges increased (+ 4.00 10^5^ cells) at the expense of viable cells displaying bulges (− 4.19 10^5^ cells).

Apparently, living cells could enter a transient, reversible morphological state that resulted in the appearance of bulges on their surface. This phenomenon was first observed after the addition of fresh substrate at 24 h of growth. It could also be observed in conditions of a substrate limitation. Taken these results together, it would seem that viable cells are able to modify their appearance rapidly in different conditions of substrate modifications in the liquid phase, whether due to an addition of substrate or an adaptation to a limitation of substrate. It reveals a reversible transition between two types of viable cell morphologies, similar but still different, between living cells displaying bulges or not. This reversible transition can occur very rapidly in case of a brutal change in the conditions of substrate concentrations in the culture medium, as in the case of substrate addition, or more smoothly, as in the case of a progressive limitation in substrate observed during the deceleration phase of the growth during usual batch cultures. This phenomenon could be observed thanks to the huge amount of data and computing power of the AI-ISM system. This one is still limited to unravel phenotypic heterogeneity based on morphological features and is not able to resolve the underlying molecular mechanisms. Nevertheless, it makes it possible to establish the bases for necessary follow-up studies on molecular heterogeneity. Future studies could be considered to better characterize (class 4—viable cells with bulges). As shown, these cells display external bulges that could be the consequence of a massive intracellular vacuolization of the cytoplasm. Even though it should be confirmed, physiological and morphological features of (class 4—viable cells with bulges) could be linked to an autophagic-like mechanism^11,22,23^. From a bioprocess management point of view, this feature of the IA-ISM system can be used advantageously to detect at an early stage a substrate concentration condition (feast or famine) likely to have an impact on the state of the cell, and adapt the feeding strategy accordingly.

In the decelerating and early stationary phases observed between 34 and 44 h of growth, the relative change in the number of dying/dead cells increased slightly, mainly caused by the increase in necrotic cells (+ 86%) and late stage necrotic cells (+ 134%), compared to apoptotic cells (+ 2%).

The following late stationary and decline phase that occurred from 44 h until 48.25 h of growth showed a massive increase in the number of dying/dead cells, due the increase in the number of apoptotic (+ 48%) and necrotic (+ 88%) cells. It is well documented that at the end of a batch culture, cells readily undergo apoptosis due to the deprivation of nutrients such as glucose, amino acids and growth factors^17,18^. Less information is available considering another main pathway for cell death, i.e. necrosis, which also impacts bioprocessing. In such a bioprocess, the presence of toxic metabolites such as lactate (0.81 g/L measured at 41 h—data not shown) and particularly ammonia (0.03 g/L measured at 41 h—data not shown) could have triggered cell to enter necrosis^48^. The high frequency data delivered by the AI-ISM system revealed that the stationary phase could first induce an increase in necrotic cells, then later only an increase in apoptotic cells. From the point of view of bioprocess management, the use of an online tool that allow to detect early the increase in the number of necrotic cells before the increase in the number of apoptotic cells during a stationary phase can also give an advantage to the operator, allowing to avoid a hardly reversible evolution of the culture towards its programmed death through apoptosis.

During the [48.25; 56 h] period, substrate limitation in the liquid phase was worsened by the shutdown of the oxygen supply. Oxygen is essential in the metabolism of hybridomas being the final electron acceptor in aerobic energy metabolism. The consequent oxygen depletion (Fig. 2A) led to a significant drop of the viability (Fig. 2C) which translated into a decrease of the number in viable cells (− 26%) and pair of cells (− 52%) and an increase in the number of apoptotic (+ 97%), necrotic (+ 110%) and necrotic late stage (+ 18%) cells. The present work shows a strong increase in the number of apoptotic cells in case of oxygen depletion, which is consistent with bibliography^18,49,50^. The increase in the number of necrotic cells is greater than expected when compared to previous work^51^ but can be explained by the effect of the oxygen depletion aggravated by the presence of toxic metabolites.

Figure 3B shows that the restart oxygen supply from 56 h was not enough to reverse the trend toward a death phase: the decrease in the number of viable cells with or without bulges (− 26% and − 41%) and pair of cells (− 56%) was still observed during the [56; 68.25 h] period. The increase in the number of apoptotic (+ 38%), necrotic (+ 20%) and necrotic late stage (+ 19%) cells was diminished compared to the relative changes measured during previous period, but still occurred.

Trying to reverse routes of cell death, the continuous phase of the studied bioprocess was started from 75.5 h. Operated at a volume of 1.5 L harvest/day, it allowed to renew the volume of culture medium completely in 3.3 days. The dilution rate of 0.0125/h was lower than the maximal growth yield of the hybridoma cell in this work, i.e. 0.04/h, preventing from a washout. The early effect of the starting of the continuous feeding phase was examined during the [75.5; 86 h] period. The number of viable cells with or without bulges still dropped (− 7% and − 14%), but their relative change compared to the previous studied period was lowered. The reason was to be found in the rapid increase in the number of cell pairs (+ 344%). Apparently, some of the cells benefited from the fresh culture medium addition and the toxic metabolites dilution, decreasing metabolic stresses^18^. Interestingly, the relative change in the number of apoptotic cells still increased (+ 9%) despite of the dilution rate, whereas the one of necrotic or necrotic late-stage cells showed a significant decline (− 45% and − 33%). With the environmental conditions used for the continuous culture operation, the hybridoma cell line utilized in this study appeared not to be able to reverse late-stage apoptosis, as it can be the case for other cell lines^18^. This physiological feature all the more justifies using a PAT tool to early detect cells taking a death route.

The results obtained during the period [86; 143.5 h] showed that the total viable and dead/dying cells was decreasing constantly (Fig. 3) with a viability range stabilized around 40%. Two more unwanted offgas clogging events occurred during this period of the bioprocess. Because viability could not be recovered, the continuous phase of the culture was stopped at 150 h of growth.

The study carried out with this new non-invasive, on-line AI-ISM technique produced cell density and viability estimates showing very high accuracy (MAPE of 6.70% and 1.68% respectively) under non-suboptimal culture conditions. Further experiments should be done using other cell types to verify the validity and robustness of the technique. In this work, it was also possible to observe a particular cell pattern, showing bulges and presenting less homogeneous texture and circular outline compared to viable cells. It was hypothesized that these bulges could be linked to intracellular vacuolization of the cytoplasm, which is a feature of the autophagy-type mechanism. These cells have been shown to respond to “feast” or “famine” environmental conditions^23^. Future studies should be envisaged to better characterize these cells, which could be studied in greater detail. From a bioprocessing point of view, these cells should be used as an early warning indication of particular culture conditions linked to substrate concentration, information that will be useful to bioprocess engineers (to better control the process) as well as scientists.

## Methods

### In situ microscope technical data and working principle

The pulsed transmitted light microscope with an objective × 40, NA 75, object field 0.16 × 0.22 mm^2^ water immersion (LOMO, St. Petersburg, Russia) is coupled to a camera CCD-size 2/3’’, 1392 × 1040 pixels corresponding to 6.6 × 8.8 mm^2^, pixel size 6.45 × 6.45 µm^2^ (A102f, Basler, Ahrensburg, Germany)^29^. A 300 µm glass fiber is combined to POF-coupled luminescence diode (DieMOUNT, Wernigerode, Germany). The frequency of evaluated camera frames is 5 per second. The size of cell-micrographs is 140 × 140 pixels, corresponding to 22.5 × 22.5 mm^2^.

Pulses for transmitted light illumination via the optical fiber are generated by the luminescence diode. The suspension is separated from the objective by a quartz-glass window positioned opposite of the fiber ending. A gap between fiber-ending and quartz-surface allows the suspension to flow through freely. Because of the spatial vicinity of the mixing impeller, it is estimated that the suspension has a time mean velocity close to the top velocity of the impeller, i.e., 0.1 m/s in this study. The cells drifting in front of the window can be imaged on a CCD without blurring thanks to the flash illumination of the luminescence diode. Cells appearing with sharply imaged details belong to the probe volume. Historically, counting via depth of field was first reported by Jean Perrin in 1909 in an experiment on the microscopic observation of an emulsion^52^. Individual cell analysis can thus be considered to study the heterogeneity of a population of cells. Only one single momentary image is obtained from a set of cells which happens to be moving through the observed probe volume during the short illumination flash. After each illumination flash, the flowing suspension immediately shifts a new cell-sample into the probe volume. Subsequent flashes will always image entirely new cell-samples. Hence, it is impossible to follow the dynamics of the morphology of one individual cell with ISM, nor its descendants.

### Cell cultivation in bioreactor, counting and viability analysis

The hybridoma cell line was a BalB/c murine lymphoid cell fused with a murine myeloma cell (DGB02/C7021—Diagast SA, Loos, France) cultured in DMEM with 4 mM l-glutamine and 4.5 g/L glucose + 10% heat inactivated fetal calf serum. The bioreactor used was a Bioflo 3000 (New Brunswick Scientific, Eppendorf, Hamburg, Germany) with a working volume of 5 L. Measured seeding viable cell density was 0.6 10^6^ cells/mL. The pH-value was controlled at 7.2 by addition of carbon dioxide. Dissolved oxygen was controlled at 30% of the saturation concentration by injection of pure oxygen. Mixing impeller was a pitched blade turbine (4 blades). Stirring was kept at 50 rpm and temperature at 37 °C. The initial cell density was approximately 4 10^5^ cells per mL. Under these conditions, a batch culture lasting 75.5 h was carried out followed by a continuous phase lasting 145 h operated at a volume of 1.5 L harvest/day. Ex situ measurements of cell concentration and viability were done with routine trypan blue dye exclusion assay^45^.

### CNN

YOLO was chosen because of its ability to detect objects with inference speeds compatible with real time applications, allowing to get an output in a few milliseconds^53^. The performance of YOLOv4 applied to ISM-images resized by default (608 × 608 pixels) allowed an inference speed superior to 30 images per second with using a conventional Nvidia GeForceGTX 1070 GPU for laptop. As ISM-sensor data were made available with a frequency of 5 images per second, real-time monitoring of cell classes became feasible, without upgrading YOLO toward its latest version (v10). The labelling of a learning database was first done. The annotation of 4000 images by two interchanging experimenters was done according to the identification rule. The database was made up of images taken by ISM in suspended cultures showing viability values ranging from 90 to 20%. The annotation step was done with using LabellImg version 1.8.4. The training phase of the object detection algorithm was then performed on a set of 3400 images. The test phase that follows was done on a set of 600 images, until the performance of the model reached a maximized confidence score. The determination of the mean average precision (mAP) value in function of the number of iteration was used for this maximization. The task of real-time object detection finally consisted in localizing objects and classifying them automatically according to a mAP value given for each object localized by the detector.

## Data Availability

The 4000-images used for the labelling phase are available in the CNRS Research Data repository, [10.57745/YSCL8Z]. The complete 848 192-image database analysed during the current study is available from the corresponding author on reasonable request.
